# A smartphone-interfaced, low-cost colorimetry biosensor for selective detection of bronchiectasis *via* an artificial neural network

**DOI:** 10.1039/d2ra03769f

**Published:** 2022-08-26

**Authors:** Mizaj Shabil Sha, Muni Raj Maurya, Muhammad E. H. Chowdhury, Asan G. A. Muthalif, Somaya Al-Maadeed, Kishor Kumar Sadasivuni

**Affiliations:** Center for Advanced Materials, Qatar University P.O. Box 2713 Doha Qatar kishor_kumars@yahoo.com; Department of Electrical Engineering, Qatar University P.O. Box 2713 Doha Qatar; Department of Mechanical and Industrial Engineering, Qatar University P.O. Box 2713 Doha Qatar; Department of Computer Science and Engineering, Qatar University P.O. Box 2713 Doha Qatar

## Abstract

Exhaled breath (EB) contains several macromolecules that can be exploited as biomarkers to provide clinical information about various diseases. Hydrogen peroxide (H_2_O_2_) is a biomarker because it indicates bronchiectasis in humans. This paper presents a non-invasive, low-cost, and portable quantitative analysis for monitoring and quantifying H_2_O_2_ in EB. The sensing unit works on colorimetry by the synergetic effect of eosin blue, potassium permanganate, and starch-iodine (EPS) systems. Various sampling conditions like pH, response time, concentration, temperature and selectivity were examined. The UV-vis absorption study of the assay showed that the dye system could detect as low as ∼0.011 ppm levels of H_2_O_2_. A smart device-assisted detection unit that rapidly detects red, green and blue (RGB) values has been interfaced for practical and real-time application. The RGB value-based quantification of the H_2_O_2_ level was calibrated against NMR spectroscopy and exhibited a close correlation. Further, we adopted a machine learning approach to predict H_2_O_2_ concentration. For the evaluation, an artificial neural network (ANN) regression model returned 0.941 *R*^2^ suggesting its great prospect for discrete level quantification of H_2_O_2_. The outcomes exemplified that the sensor could be used to detect bronchiectasis from exhaled breath.

## Abstract 2

Detection of bronchiectasis from exhaled breath.

## Graphical abstract

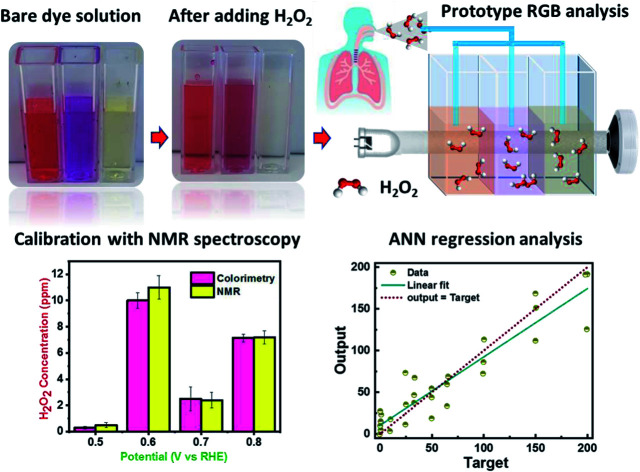

## Introduction

Exhaled Breath (EB) contains numerous bioproducts that act as biomarkers and reflect well-being. Biomarkers represent the physiological and enzyme reactions occurring in the body, and their quantification in EB can help detect disorders.^1^ Compared to usual clinical tests, breath monitoring is a non-invasive method; hence, the chances of infection are less and suitable for long-term clinical monitoring.^2,3^

Linus Pauling first proposed breath detection in 1970 and successfully detected around 250 bioproducts in the EB by gas chromatography. Breath analysis is done by either monitoring EB in the gas phase or tracking it in the aqueous phase.^4,5^ Hydrogen peroxide (H_2_O_2_) is an excellent example and one of the crucial markers in treating numerous diseases.^6^ The EB of people with asthma, systemic inflammation, chronic obstructive pulmonary disease (COPD), acute respiratory distress syndrome (ARDS), bronchiectasis, systemic sclerosis, cystic fibrosis, systemic inflammation, uremia, and pneumonia have elevated levels of H_2_O_2_ (see Table 1). The H_2_O_2_ levels for these diseases vary between 10 nmol L^−1^ to 10 μmol L^−1^.^7^

**Table 1:** Biomarkers detected in EB correlate with various pulmonary diseases.^20^

Diseases	Biomarkers
Smoking	8-Isoprostane, H_2_O_2_
Chronic obstructive pulmonary disease	Serotonin, H_2_O_2_, 8-isoprostane, cytokines
Asthma	Leukotrienes, H_2_O_2_, 8-isoprostane, nitrotyrosine thiobarbituric acid-reactive products
Bronchiectasis	H_2_O_2_
Chronic bronchitis	Leukotrienes
Cystic fibrosis/idiopathic pulmonary fibrosis	Nitrite, IL-8, H_2_O_2_, 8-isoprostane
Acute respiratory distress syndrome	H_2_O_2_, 8-isoprostane, PGE_2_

Bronchiectasis is a chronic inflammatory lung illness marked by permanent bronchial dilation. Airway secretions include high quantities of proinflammatory cytokines, and neutrophils are the most common cells in the airway lumen. Bronchial damage occurs in people with bronchiectasis due to neutrophil inflammatory agents generated in response to bacterial infection.^8^

Inflammatory cells such as neutrophils, eosinophils, and activated macrophages produce a lot of superoxide anion (O_2_^−^), which is disputed from hydrogen peroxide either spontaneously or with the help of enzymes. H_2_O_2_ appears to be a significant reactive oxygen species that cause cellular harm and produces other reactive oxygen species such as hydroxyl radicals and lipid peroxidation products.^9^

Furthermore, H_2_O_2_ is a key compound in the food industry, chemical industry, laboratory, environmental, and pharmaceutical analysis.^10^ Conventional techniques to quantify H_2_O_2_ include spectrophotometer, chemiluminescence, electrochemistry, spectro fluorometry, and surface plasmon resonance. These techniques are of high cost and require heavy-duty instruments. Even though enzyme-based electrochemistry has high selectivity and sensitivity, the difficulty in immobilizing and stabilizing enzymes limits their use.^11–13^

Jeong-Hyeop *et al.* suggested a biosensor that works by the electrocatalytic activity of horseradish peroxidase (HRP)-encapsulated protein nanoparticles (HEPNP) for the detection of H_2_O_2_.^14^ Ali-saad Elewi *et al.* also developed a biosensor based on hemoglobin immobilized on a screen-printed carbon electrode (SPCE).^15^ Despite these advancements, there is still a pressing need to create simple, cost-effective, non-invasive, and quick H_2_O_2_ quantification methods.^16^

Among all the analytical procedures, the colorimetric assay has shown tremendous attention due to its simplicity, speed of execution, high sensitivity and selectivity. Additionally, the colorimetric imaging device overcomes these problems of uneconomically tedious and complex procedures. Further, a smoothly miniaturized alternative was developed where light is separated into a set of color spaces, primarily red, green, and blue (RGB).^17^ However, color space-assisted colorimetric analysis significantly impacts ambient light conditions and camera optics. This can be addressed by controlling and confining the light conditions while characterizing the sample.

Further, advanced algorithms such as machine learning can be adopted for statistical analysis. With its powerful utilities like self-learning from the data and automated decision-making, machine learning is a dominant method for quantitative evaluation. Combining machine learning with colorimetry will enhance its adaptability and flexibility to emerging frameworks such as Internet of Things (IoT) systems.^18,19^

This study has adopted a three-dye system for more accurate and precise colorimetry sensing. An IoT-enabled detection unit with controlled light conditions can detect RGB color space values within one minute and impart supplementary H_2_O_2_ data. The detection results are displayed on mobile with designated RGB parameters correspond to the specific concentration of the H_2_O_2_. Further, we have explored the machine learning algorithm to estimate H_2_O_2_ levels based on the RGB value extracted from the sensor prototype. Our research will pave the way for novel, handy, compact, and versatile sensors and, in turn, to identify and analyze H_2_O_2_ in the exhaled breath, which serves as an essential biomarker for detecting body bronchiectasis.

## Materials and methods

### Materials and instruments

Hydrogen peroxide (30% w/w), acetonitrile, acetone (99.5% w/v), benzene (37% w/v), ammonia (25%), chloroform, deuterium oxide, eosin blue (2% w/v), formaldehyde (99% w/v), methanol, porphyrin solution, potassium iodide, potassium permanganate (99% w/v), silver nanowire, soluble starch powder (*M*_w_ 692.661 g mol^−1^) and toluene (38% w/v) were purchased from Sigma-Aldrich. All the chemicals used in the experiment were of analytical grade.

### Methods

Starch solution of 0.1 mM was prepared by mixing corn starch powder in double distilled (DI) water. The mixture was heated to dissolve starch completely. Thereafter, a drop of iodine solution was added in 10 mL of 0.1 mM starch solution, that turns the solution in blue color. Further, to enhance the H_2_O_2_ sensing properties, a drop of silver nanowire was added in the as prepared starch-iodine solution.^21^

The solutions of potassium permanganate (KMnO_4_) and eosin blue were also prepared in DI water at a concentration of 3 mM. The pH effect of the test analyte with eosin blue, KMnO_4_ and silver nanowire dispersed starch iodine (SI) dyes was analyzed in acidic (2, 4, 6), neutral, and basic (9, 12) solutions. Each dye solution (10 mL) was added with 1 mL of 0.3 ppm H_2_O_2_ solution in the adjusted pH. Changes in color and corresponding response time were observed. Dyes solutions were then characterised by UV-visible spectroscopy technique. The detection limit of the dyes was monitored in the range of 0.003 to 5 ppm concentrations of H_2_O_2_ solution and temperature effect was studied. Selectivity analysis was performed using 0.03 ppm of H_2_O_2_, formaldehyde, acetone, nitric oxide, ammonia, toluene and benzene at room temperature.

### Characterization

A Biochrom UV-vis Spectrophotometer (scanning range of 190–1100 nm) was used for characterization. Here a scanning range of 300–750 nm with a medium scan speed was adapted. Bruker Advance III 500 MHz spectrometer (Bruker Bio spin, Rheinstetten, Germany) with a BBFOPLUS Smart Probe equipped with a Bruker Automatic Sample Changer was used to perform NMR spectroscopy studies (B-ACS 120).

### IoT-based sensor prototype

**Fig. 1:**
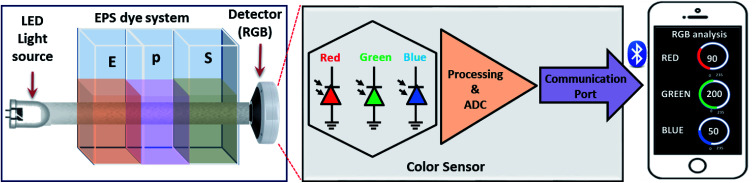
Fabricated smartphone-assisted sensor prototype.

For color space-based analysis, a sensor unit was fabricated using the 3D printer (QIDI 3D printer). The sensor prototype offered controlled ambient light conditions and optics during color space-based H_2_O_2_ quantification. The sensor prototype has three independent regions, *i.e.*, a light source zone, sample zone, and detection zone, as shown in Fig. 1. The light source consists of white light LED. The sample zone with three cuvettes consisting of individual eosin blue, KMnO_4_ and SI (EPS) dye solutions are placed sequentially in each section. The light source, sample zone, and detector are aligned horizontally to increase the reliability of the prototype. The light from the LED falls on the cuvettes containing EPS dye solution, and the detector intercepts light transmitted from the sample zone. The detector examines the light that has been transmitted, and light is divided into red, green and blue color spaces. The results are communicated to the screen *via* the Bluetooth technique.

### Validation of H_2_O_2_ measurements

To evaluate the applicability of the proposed sensor prototype, comparisons were made concerning heavy-duty instruments like the NMR spectrometer.^4^ The solution employed in the NMR experiments of H_2_O_2_ breakdown contains 75% (v/v) acetonitrile, and the rest is methanol. 3.1 mL of solvent, 5.0 mL of H_2_O_2_, and 10 mL of chloroform were combined in a 5 mL reaction vial, and 463 mL of this mixture was placed in a precision NMR tube. Before starting an experiment, an NMR tube without the catalyst was placed in the NMR spectrometer to lock the field and shim the magnet. After the NMR spectrometer was set up, 37 L of a 1.0 mM porphyrin solution was injected into the reaction mixture's NMR tube. An NMR tube containing 50 percent deuterium oxide and 50 percent water was shaken to mix the sample. The spectrometer was then allowed to lock onto the sample after the NMR tube containing the reacting sample was inserted.

## Results and discussion

To fabricate the extremely sensitive colorimetric VOC sensor, H_2_O_2_ solution (concentration ranging from 0.003–5 ppm) was added to the dye solutions in an acidic, basic, and neutral media. Any obvious color changes were monitored. Response time, pH impact, temperature effect, concentration effect, and the dyes' selective nature were also investigated and assessed.

Table 2 represents the list of notations for the study. Here *x* stands for specific pH, *y* stands for a specific temperature, and *z* stands for biomarker concentration.

**Table 2:** List of notations used

Dyes	Dye indication	pH(*x*), Temperature (*y* in °C), biomarker (H_2_O_2_ (H))-concentration (*z* in ppm) indication
KMnO_4_	KM	KM(PxTyHz)
Eosin blue	EB	EB(PxTyHz)
Starch-iodine	SI	SI(PxTyHz)

### Response time and pH effect

**Fig. 2:**
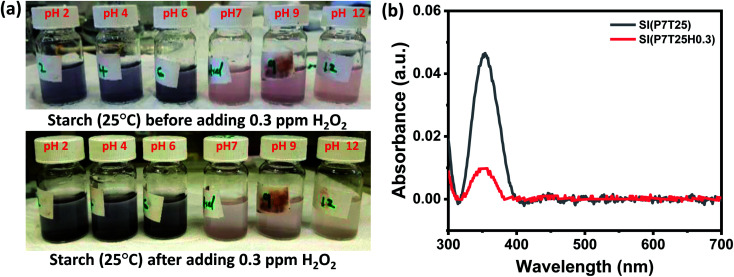
SI dye response toward H_2_O_2_. (a) pH adjusted SI dye solution before and after adding 0.3 ppm H_2_O_2_ solution at room temperature. (b) (b) Absorbance spectra of neutral SI dye solution before and after adding 0.3 ppm H_2_O_2_.

H_2_O_2_ was detected using SI, eosin blue, and KMnO_4_ dye solutions with pH 2, 4, 6, 7, 9, and 12. At room temperature, 0.3 ppm of H_2_O_2_ in 1 mL DI water was added to the dye's solution. The dyes' response time was calculated by noting the time between the addition of H_2_O_2_ and the visible color change noticed in the dye's solution. In H_2_O_2_ detection analyses using the SI dye, the color shift was visible in neutral and basic solutions but not in the acidic media. The color shift in the SI dye solution after adding 0.3 ppm H_2_O_2_ is shown in Fig. 2a. From Fig. 2a, it can be inferred that the neutral and basic starch dye solution color tends to diminish and turns slight blue with H_2_O_2_. Herein, irrespective of pH value, starch dye response time decreases with increased H_2_O_2_ concentration. In all the pH-adjusted solutions, the estimated response time for 5, 3, 0.3, and 0.03 ppm H_2_O_2_ was nearly 6 min, 8 min 22 s, 12 min 16 s, and 20 min, respectively. It can be inferred that the redox reaction between potassium iodide and H_2_O_2_ followed by the color reaction of SI could be finished within 20 min for H_2_O_2_ concentration as low as 0.03 ppm. In the acidic medium more number of H^+^ ions are available, as a result most of the iodine ions are converted to the iodine followed by the formation of the complex compound that gives the blue color. Thus the addition of H_2_O_2_ in the acidic dye medium only intensify the blue color and not much change visible color change is observed. However, with increase in the pH from neutral to basic the H^+^ ion concentration decreases which decreases the formation of the iodine ions and formation of complex compound respectively. As a result, faint pink color of solution is observed. Thus, addition of H_2_O_2_ catalysis the formation of the iodine in the neutral and basic medium that results in the appearance of slight blue color. Eqn (1) and (2) gives the kinetics of the response of the SI dye solution system and H_2_O_2_.

1 H_2_O_2_ + 2I^−^ + 2H^+^ → I_2_ + 2H_2_

2 *n*I_2_ + 6*n*(C_6_H_10_O_5_) → 2*n*(C_18_H_30_O_15_I)

Here, H _2_O_2_ liberated iodine from potassium iodide (eqn (1)), followed by the reaction of starch and iodine, resulting in a complex classic response (eqn (2)).^22^

**Fig. 3:**
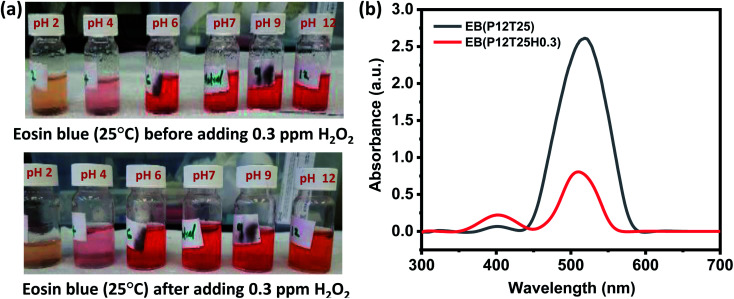
Eosin blue dye response toward H_2_O_2_. (a) pH adjusted EB dye solution before and after adding 0.3 ppm H_2_O_2_ solution at room temperature. (b) Absorbance spectra of pH 12 EB dye solution before and after adding 0.3 ppm H_2_O_2_ solution.

For the H_2_O_2_ assay in eosin blue solution, the visible color change was noticed in pH 12 solution for all H_2_O_2_ concentrations, *i.e.*, 0.03–5 ppm. Fig. 3a shows the color change in the EB solution after adding 0.3 ppm H_2_O_2_. An apparent color change from red to orange is observed in the dye solution of pH 12. The corresponding absorbance curve of pH solution before and after adding H_2_O_2_ is shown in Fig. 3b. The response time of pH 12 solution increased with a decrease in the concentration of H_2_O_2_ and was estimated to be 6 min, 8 min, 15 min, and 25 min for 5, 3, 0.3, 0.03 ppm test solution, respectively. In eosin blue dye, the presence of polycyclic aromatic ring produces the most intense peak at ∼518 nm. Therefore, change of color indicates the degradation of the polycyclic aromatic rings. In neutral and basic aqueous solution, eosin blue (EB) exist as EB^2−^(aq), while in week and strong acidic medium eosin blue forms HEB^−^ (aq) and H_2_EB (aq), respectively. Thus, with the formation of ionic compound, there are more chances of eosin blue degradation in week acidic, neutral and basic medium. Moreover, H_2_O_2_ is more stable in the acidic medium compared to the basic medium. Thus, in acid medium H_2_O_2_ doesn't undergo auto degradation to produce free radical that can react with EB and perform dye degradation. Whereas, in the pH 12 solution, H_2_O_2_ undergoes auto degradation to form HO˙ radical, as given by eqn (3) and eqn (4).

3 H_2_O_2_ → 2H^+^ + HO_2_^−^

4 H_2_O_2_ + HO_2_^−^ → HO_2_˙ + HO˙ + OH^−^

The generated HO˙ radicals react with eosin blue and results in the degradation of the dye that is represented by the degradation of the dye color in pH 12 dye solution, as shown in Fig. 3a.

**Fig. 4:**
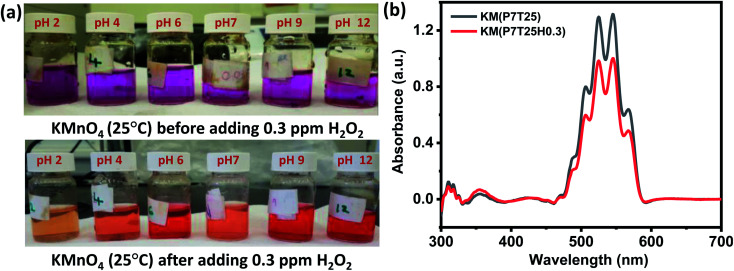
KMnO_4_ dye response toward H_2_O_2_. (a) pH adjusted KMnO_4_ dye solution before and after adding 0.3 ppm H_2_O_2_ solution at room temperature. (b) Absorbance spectra of neutral KMnO_4_ dye solution before and after adding 0.3 ppm H_2_O_2_ solution.

Interestingly, the H_2_O_2_ assay in KMnO_4_ dye solution exhibited a prominently visible color change in all pH mediums compared to SI and EB dye solutions. In addition, the color variation of the solution from violet to light orange and violet to deep orange after adding H_2_O_2_ is visually observed irrespective of the concentration of the test solution. The color change in the KMnO_4_ solution after adding 0.3 ppm H_2_O_2_ is shown in Fig. 4a. At concentrations of H_2_O_2_ as low as 0.3 ppm, the apparent color change may be identified, providing a simple method for detecting H_2_O_2_ with the naked eye.

Like other dyes, the response time of KMnO_4_ increases with a decrease in the test solution concentration. Compared to the acidic and basic medium, the neutral KMnO_4_ solution exhibits high assay performance with a 1, 7, 13, and 27 s response time for 5, 3, 0.3, and 0.03 ppm, respectively. Redox reaction of KMnO_4_ is given by eqn (5).

5 5H_2_O_2_ + 6H^+^ + 2KMnO_4_ → 5O_2_ + 2Mn^2+^ + 8H_2_O + 2K^+^

Further in the study, for SI and KMnO_4_ dye solution we have opted for the neutral dye medium, since it removes the pH adjustment step and simplifies application of the dye in the fabricated sensing prototype system.

### Concentration effect and limit of detection

**Fig. 5:**
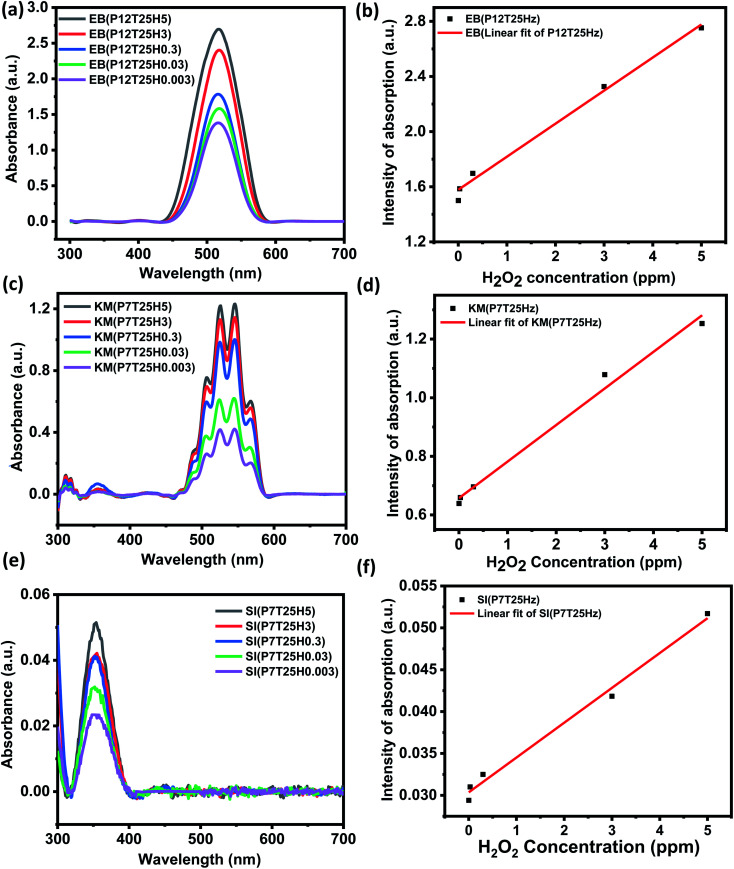
Dyes sensitivity towards H_2_O_2_ detection. (a and b) UV-vis absorption curve with change in H_2_O_2_ concentration and corresponding calibration plot of eosin blue dye, respectively. (c and d) UV-vis absorption curve with H_2_O_2_ concentration shift and corresponding calibration plot of KMnO_4_ dye, respectively. (e and f) UV-vis absorption curve with varying H_2_O_2_ concentration and corresponding calibration plot of starch dye, respectively.

The UV-vis study was carried out to determine the sensitivity of the colorimetric EPS system by adjusting the H_2_O_2_ content in the SI (pH 7), KMnO_4_ (pH 7) and EB (pH 12) dye solutions from 0.003 to 5 ppm. Fig. 5 illustrates the UV-vis absorbance plot of the dyes with changes in H_2_O_2_ from 0.003–5 ppm and the corresponding calibration for determining the dye's LOD towards H_2_O_2_. The LOD was estimated using the 3*σ*/*m* criterion after the calibration curve was plotted using the dye's peak absorbance at a certain wavelength. Where *m* is the calibration plot's slope and *σ* is the intercept's standard deviation. Fig. 5a and b show the UV-vis plot of the EB dye as a function of H_2_O_2_ concentration and calibration cure. As the concentration of H_2_O_2_ rises, the absorbance also rises (Fig. 5a). The calibration curve was plotted using the dye's peak absorbance at 518 nm for various H_2_O_2_ concentrations. The linear fitting was performed in the range of 0.003–5 ppm H_2_O_2_ and estimated LOD of EB was ∼0.011 ppm (Fig. 5b), *y* = (2.3984) *x* + (1.57804 ± 0.00906); *R*^2^ = 0.94159. Like EB, an increase in absorbance with an increase in H_2_O_2_ ppm level was noticed for KMnO_4_ dye (see Fig. 5c). The calibration curve was plotted from 0.003–0.3 ppm concentration by considering the absorbance at 525 nm. Fig. 5d shows the calibration curve of KMnO_4_ dye in the linear range of 0.003–5 ppm. The linear fit to the data revealed an LOD of ∼0.025 ppm towards H_2_O_2_ sensing by KMnO_4_ dye, *y* = (0.12484) *x* + (0.6571 ± 0.001044); *R*^2^ = 0.98944). SI dye also exhibited a linear relationship with the concentration, as shown in Fig. 5e. With an increase in H_2_O_2_ concentration, the absorbance also increased. The calibration curve was plotted using the peak dye absorbance at 354 nm for various H_2_O_2_. The linear fitting was done in the range of 0.003–5 ppm H_2_O_2_ and the predicted LOD of the starch dye was 0.044 ppm, *y* = (0.0416) *x* + (0.03035 6.109 × 10^−4^); *R*^2^ = 0.9906). According to the sensitivity study, the EPS dye system has a high sensitivity to H_2_O_2_, with a detection limit of 0.011 ppm.

### Temperature effect

**Fig. 6:**
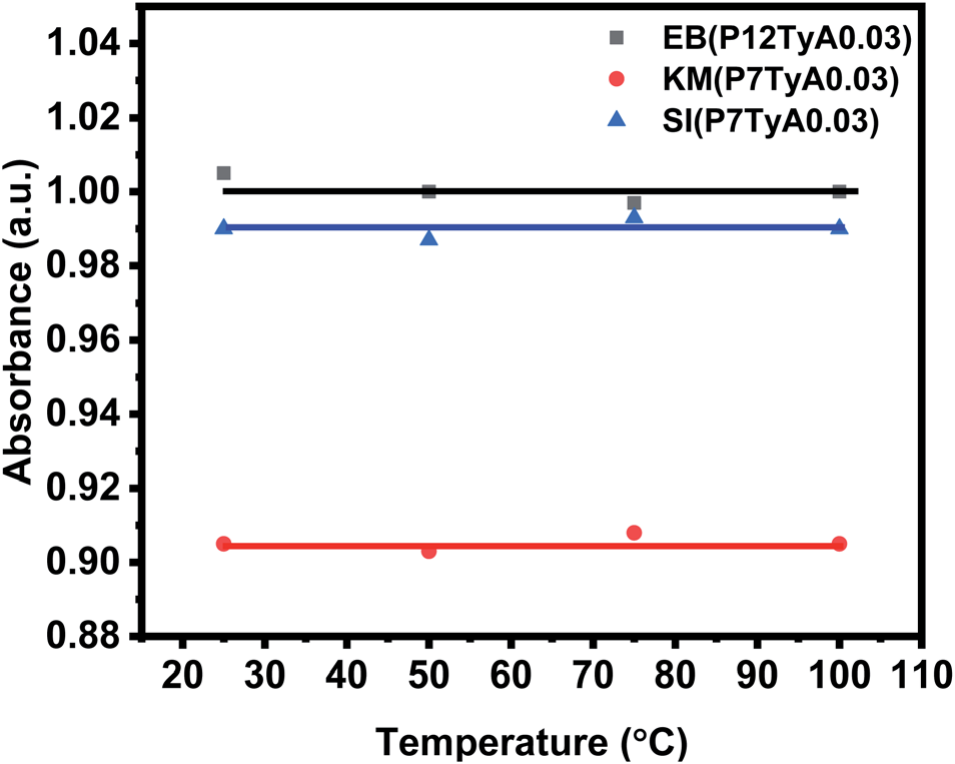
Temperature effect on H_2_O_2_ detection in EPS dye solutions system.

Even though they are critical, shelf life and stability qualities are frequently understudied or ignored in the literature. At an elevated temperature, instability or aging can be hastened. To investigate this parameter, dye solutions were heated at different temperatures, *i.e.*, 25 °C, 50 °C, 75 °C and 100 °C. The dye solutions were given 1 mL of the test solution, with a concentration of 0.03 ppm. As demonstrated in Fig. 6, the absorption intensity of the EPS dye solution remained nearly constant regardless of temperature change. This showed that the EPS dye system is temperature stable, which is important for the colorimetric sensor.

### Selectivity analysis

**Fig. 7:**
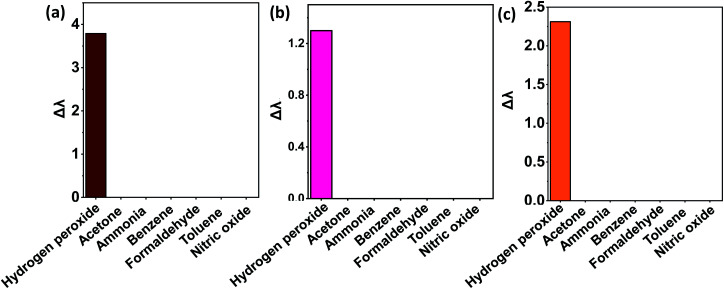
Selectivity analysis of biomarkers in different dye solutions (a) Eosin Blue dye solutions (b) KMnO_4_ dye solution (c) SI dye solution.

Control studies with potential interfering biomarkers in breath such as acetone, benzene, ammonia, formaldehyde, toluene, and nitric oxide were done in EPS dye solution to evaluate the selectivity of H_2_O_2_ detection. The H_2_O_2_ and possible interfering analytes concentrations were both 0.03 ppm. The dye's selectivity for H_2_O_2_ was determined using UV-vis analysis and eqn (6).6
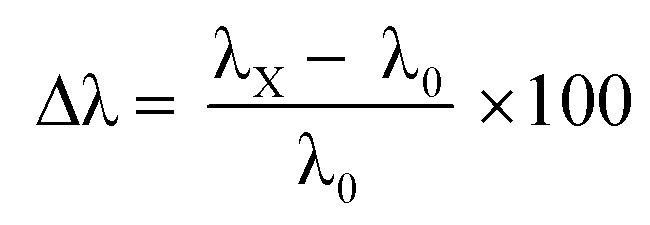
where *λ*_*x*_ is the analyte's unique peak absorbance wavelength, and *λ*_0_ is the wavelength of the blank solution with high absorbance. The *λ*_*x*_ value is measured at pH 12 for EB and a neutral solution for KMnO_4_ and SI dye. In the case of all three dyes, it was observed that only H_2_O_2_ could induce a shift in UV-vis peak absorbance, as shown in Fig. 7. According to these findings, other interfering compounds did not compete with the H_2_O_2_ chemo-indicator for colorimetric detection. As a result, the EPS dye system can be used to create a highly specific colorimetric sensor for H_2_O_2_ molecules.

### Real-time application of the proposed sensor and evaluation

A portable prototype device with full functions for detecting hydrogen peroxide was constructed. Employing the three dyes as sensing elements, the sensor prototype showed a unique set of RGB values upon exposure to 0.03 ppm hydrogen peroxide (Table 3).

**Table 3:** Analysis of H_2_O_2_-EPS mixture using Sensor Prototype

H_2_O_2_-EPS Mixture	RGB values obtained for different light source			
	Red light	Blue light	White light	Green light
EPS	255,0,0	103,0,255	255,0,226	19,88,50
E(H_2_O_2_) PS	116,0,8	33,0,109	124,0,109	22,44,7
EP(H_2_O_2_) S	103,0,5	0,38,90	82,94,101	0,94,4
EPS(H_2_O_2_)	179,0,9	36,0,255	175,47,239	0,87,39
E(H_2_O_2_) P(H_2_O_2_) S	175,0,3	32,50,109	165,118,98	0,130,0
E(H_2_O_2_) PS(H_2_O_2_)	181,07	42,6,197	181,56,184	0,89,30
EP(H_2_O_2_) S(H_2_O_2_)	123,0,5	20,34,84	124,92,76	0,104,0
E(H_2_O_2_) P(H_2_O_2_) S(H_2_O_2_)	121,0,3	27,34,82	116,90,73	0,97,0

**Fig. 8:**
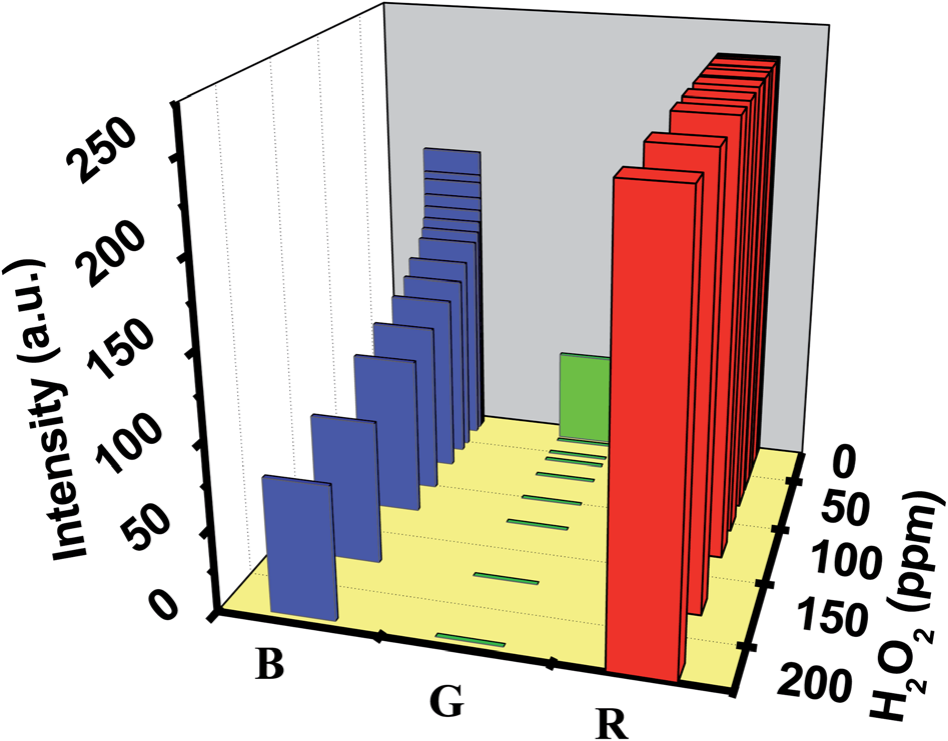
3D plot, representing RGB value corresponding to the H_2_O_2_ level.

Employing the EPS dye system, the sensor prototype displayed a unique set of RGB values when exposed to diverse H_2_O_2_ concentrations ranging from 0.001 to 200 ppm. The sensor prototype displayed a unique set of RGB values. Fig. 8 shows the 3D plot, representing the RGB value corresponding to the H_2_O_2_ level. A decrease in the intensity of the ‘B’ color space value is observed with an increase in the H_2_O_2_ concentration.

Each RGB value corresponds to a unique concentration of H_2_O_2_ that can be correlated to clinical information for quantifying bronchiectasis in the human body.

**Fig. 9:**
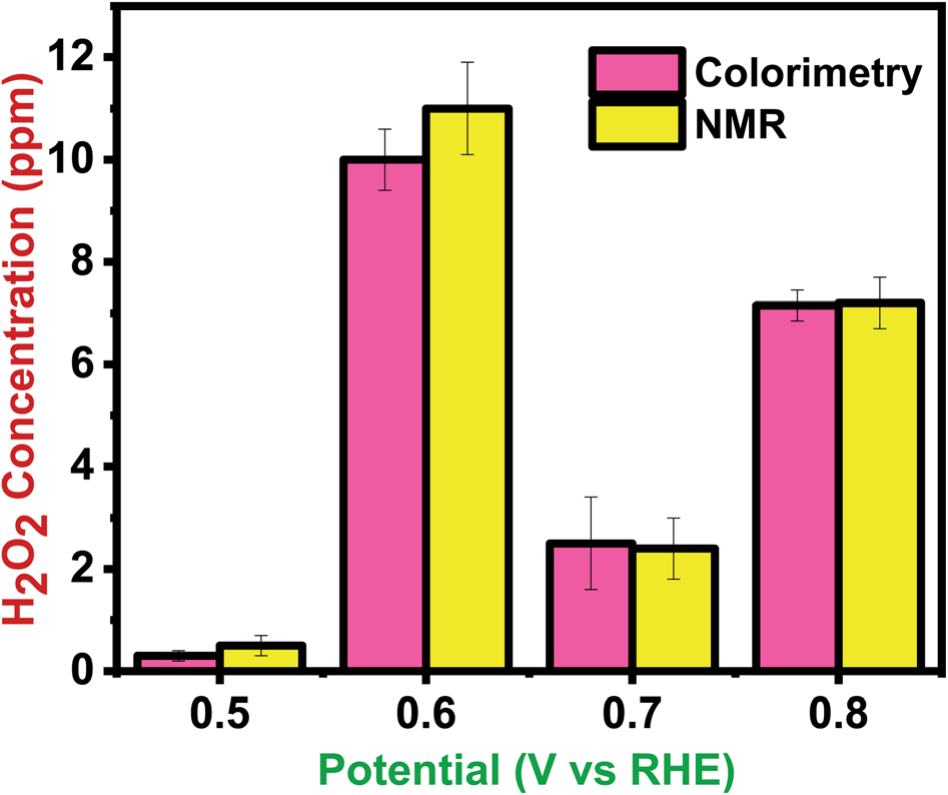
Comparison of NMR data and colorimetric data for quantification of H_2_O_2._

To investigate the accuracy of the proposed colorimetric method, the sensor device was calibrated against the NMR spectroscopy technique. Fig. 9 compares the efficiency of NMR and colorimetric data for H_2_O_2_ detection. The quantification data at the potential range of 0.5–0.8 V shows the concentration in the range of 0.5–7.2 ppm in NMR and 0.3–7.15 ppm in the sensor prototype. The colorimetry and NMR integration results were mostly in accord, with some overestimated related compounds due to peak overlap, integral errors, and a lower estimate for the target chemical. The study shows that this innovative method for colorimetric detection and quantification of H_2_O_2_ is accurate for chemical analysis. Moreover, according to the cost comparison between NMR and colorimetry, it was shown that this IoT coupled sensing prototype is cost-effective as opposed to the high-cost NMR analysis. Furthermore, unlike NMR, this colorimetric technique would yield results in seconds rather than hours.

### Artificial neural network (ANN) regression

**Fig. 10:**
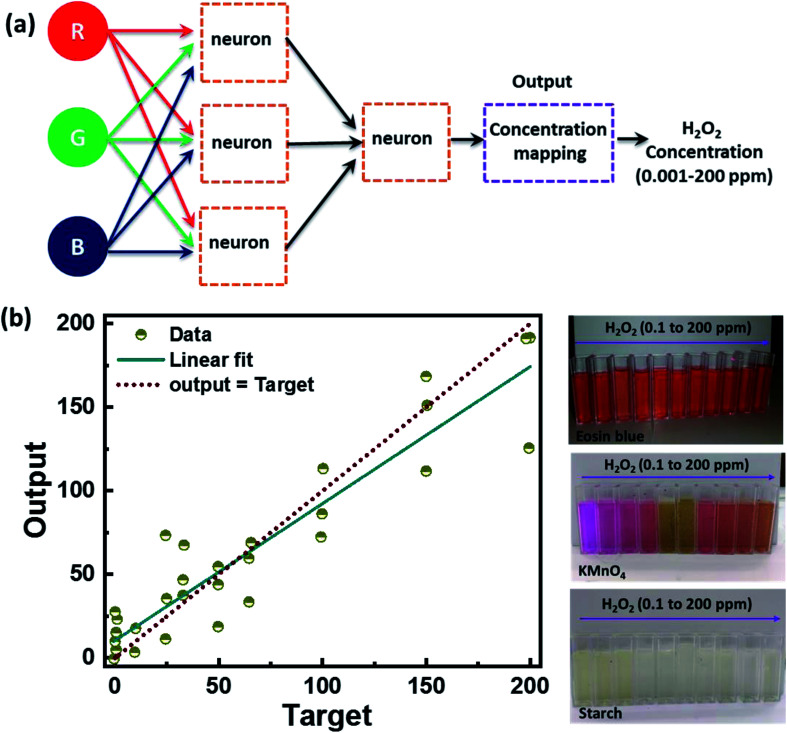
(a) Flow diagram of regression analysis using artificial neural network (ANN) regression algorithm. (b) The ANN machine learning algorithm plot shows the linear fit between the target and output value (left) and the change in color of the colorimetric dye solution after detecting H_2_O_2_ in the range of 0.1 to 200 ppm level (right).

Further, we have also analyzed the RGB color space value extracted from the sample under white light illumination and employed artificial neural network (ANN) regression machine learning algorithms to identify the corresponding H_2_O_2_ level. We used 15 different concentrations of H_2_O_2_ ranging from 0.001 ppm to 200 ppm at different temperatures of 25 °C and 50 °C. A total of thirty sets of readings were obtained, out of which twenty-five were used for training the model, and five were used for testing. Fig. 10 shows the ANN machine learning algorithm's output results and the color change of colorimetric dye solution detecting H_2_O_2_ at 0.1 to 200 ppm-level. Fig. 10a represents the flow diagram of the ANN machine learning algorithm. The algorithm is divided into sub-module layers of the neurons where the neuron gets the H_2_O_2_ concentration and RBG value as three input training parameters. The output of the three neurons in the sub-modules is given as input to the neuron in another submodule layer. The output from the neuron is then used for the concentration mapping, and the final output gives the concentration of the H_2_O_2_ at the ppm-level. Fig. 10b shows the ANN machine learning algorithm plot with a linear fit between the target and the output value and a change in color of colorimetric dye solution after detecting H_2_O_2_ in the range of 0.1 to 200 ppm-level. The ANN model exhibited ∼94% accuracy, indicating that the proposed method fused with machine learning is a great prospect for discrete level quantification of H_2_O_2_. The results demonstrate the colorimetry's potential for detecting Volatile Organic Compounds (VOCs) in our exhaled breath. It can also be used in the biomedical, nuclear, food, *etc.*

## Conclusion

This study examined a new implementation of a colorimetry-based non-invasive, low-cost detection method to classify hydrogen peroxide (H_2_O_2_) levels. A portable 3D printed sensing device offering controlled light and optic conditions were designed for detection. Three dyes eosin blue, potassium permanganate (KMnO_4_) and starch-iodine having Silver Nanowire (SI), were used as sensing elements. All the dyes exhibited promising results in detecting H_2_O_2,_ and the dye samples were characterized by UV-vis spectroscopy. The estimated detection limit for eosin blue, KMnO_4_ and SI dye solution was 0.011 ppm, 0.025 ppm and 0.044 ppm, respectively. The dyes apprehended high selectivity for determining H_2_O_2_ in the presence of other interfering biomarkers. Using the EPS dye system, the developed sensor device presented red, green and blue (RGB) color space values correlated with the H_2_O_2_ concentration. The sensor prototype displayed a high resemblance with the NMR spectroscopy, where the developed sensor device estimated 0.5–7.2 ppm H_2_O_2_ level in NMR was 0.3–7.15 ppm.

Further, a machine learning classifier was trained using the RGB value obtained from the sensor prototype under white light illumination. This improved the robustness of the detection platform, and the adopted algorithm displayed ∼94% accuracy, indicating that the proposed colorimetric method fused with machine learning is a great prospect for discrete level quantification. Moreover, the reported sensor device can be versatilely extended to quantify various targets as long as suitable colorimetric agents are available. The successful applications of this novel approach have great potential as a visual sensor platform in the biomedical field, food industry, chemical industry, and environmental and pharmaceutical analysis.

## Author Contributions

Mizaj Shabil Sha: synthesis, experimentations, real sample analysis, investigation, data organization and writing – original draft. Muni Raj Maurya: real sample analysis, sensing mechanism interpretation and Writing – review & editing. Muhammad E.H. Chowdhury: writing – Review & Editing. Asan G. A. Muthalif: Software, Validation. Somaya Al-Maadeed: data organization and writing – review & editing. Kishor Kumar Sadasivuni1 (Corresponding author): conceptualization, supervision, writing – review & editing, resources, funding acquisition and project administration.

## Conflicts of interest

The authors declare that they have no conflict of interest.

## Supplementary Material

RA-012-D2RA03769F-s001
